# Case Report: A patient presenting primarily with psychosis of NPSLE treated with telitacicept, corticosteroids, and immunosuppressants

**DOI:** 10.3389/fimmu.2025.1626721

**Published:** 2025-07-11

**Authors:** Wei Xie, Peijue Chen, Lili Luo

**Affiliations:** The Third Hospital of Mianyang, Sichuan Mental Health Center, Mianyang, China

**Keywords:** neuropsychiatric systemic lupus erythematosus, telitacicept, biologics, treatment, case report

## Abstract

Therapeutic strategies for neuropsychiatric systemic lupus erythematosus (NPSLE) primarily target underlying pathogenic mechanisms and typically include corticosteroids, immunosuppressants, anticoagulation, and adjunctive treatments. B-cell–targeted biologics are an emerging option for NPSLE. Although telitacicept is approved for systemic lupus erythematosus (SLE) treatment in China, evidence supporting its use in NPSLE remains limited. This case report presents a 20-year-old female diagnosed with NPSLE who exhibited psychotic symptoms as the predominant manifestation and was treated with telitacicept, corticosteroids, and immunosuppressants. During the treatment period, her psychiatric symptoms remained in remission without infections or adverse events. Her disease activity score (SLEDAI-2000) declined from 23 to 2.

## 1 Introduction

Systemic lupus erythematosus (SLE) is a complex autoimmune disease. When it involves the central or peripheral nervous system, it is classified as neuropsychiatric SLE (NPSLE), which can present as the initial or sole clinical manifestation of SLE (1). In 1999, the American College of Rheumatology (ACR) defined 19 neuropsychiatric syndromes associated with SLE, including five psychiatric manifestations: acute psychotic state, anxiety disorder, cognitive dysfunction, affective disorder, and psychosis (2). Reported prevalence rates for NPSLE vary widely, ranging from 37% to 95%, which usually correlates with SLE activity (3), and NPSLE is effective with immunosuppressive therapy (4). Patients with NPSLE often experience a marked decline in quality of life, greater than that observed in other chronic conditions (5) and face increased mortality rates, with infection identified as the leading cause of death (37.5%) (6). Telitacicept is a recombinant fusion protein that binds and neutralizes B-lymphocyte stimulator (BLyS) and a proliferation-inducing ligand (APRIL), thereby inhibiting the development and survival of plasma cells and mature B cells. It has been approved by the National Medical Products Administration (NMPA) for the treatment of SLE in China (7). Here, we report a case of NPSLE primarily presenting with psychosis, who was treated with telitacicept, corticosteroids and immunosuppressants (mycophenolate mofetil, hydroxychloroquine). The patient showed marked clinical improvement over the 5-month follow-up, without developing infections or other adverse events.

## 2 Case description

The patient was a 20-year-old female who presented with neuropsychiatric symptoms, initially managed at another hospital nine months before admission. At presentation, she exhibited fever, auditory hallucinations, persecutory delusions, visual distortions (metamorphopsia), insomnia, and poor sleep quality. Laboratory tests conducted at the other hospital revealed leukopenia (WBC 3.31×10^9^/L), mild anemia (Hb 105 g/L), and strong positivity for antinuclear antibodies (ANA, 1:10000), anti-Smith (+++), anti-dsDNA (+++), and anti-nRNP (++). Complement levels were markedly reduced (C3: 49 mg/dl, C4: <7 mg/dl). Based on these findings, she was diagnosed with SLE with central nervous system and hematologic involvement. Initial treatment included prednisone, hydroxychloroquine, cyclophosphamide (cumulative dose 0.8 g), intrathecal methotrexate and dexamethasone, rivaroxaban, levetiracetam, and olanzapine. During therapy, she developed seizures and impaired olfaction, taste, and tactile perception. Over one month, her psychiatric and cognitive symptoms gradually improved, prompting discontinuation of medications.

Three days prior to admission to our institution, she experienced a recurrence of psychiatric symptoms, including delusions of victimization and reference, incoherent speech, irritability, and impulsive behavior, triggered by familial criticism. Family history revealed a maternal grandmother with a psychiatric illness. Physical examination showed alopecia and mild bilateral lower-limb pitting edema. Neurological examination and electroencephalography were unremarkable. The Scale for the Assessment of Positive Symptoms (SAPS) and Scale for the Assessment of Negative Symptoms (SANS) were used to evaluate psychosis. SAPS yielded a score of 71, with prominent positive symptoms such as delusions, bizarre behavior, and positive formal thought disorder. SANS scored 25, with no significant negative symptomatology.

**Figure 1:**
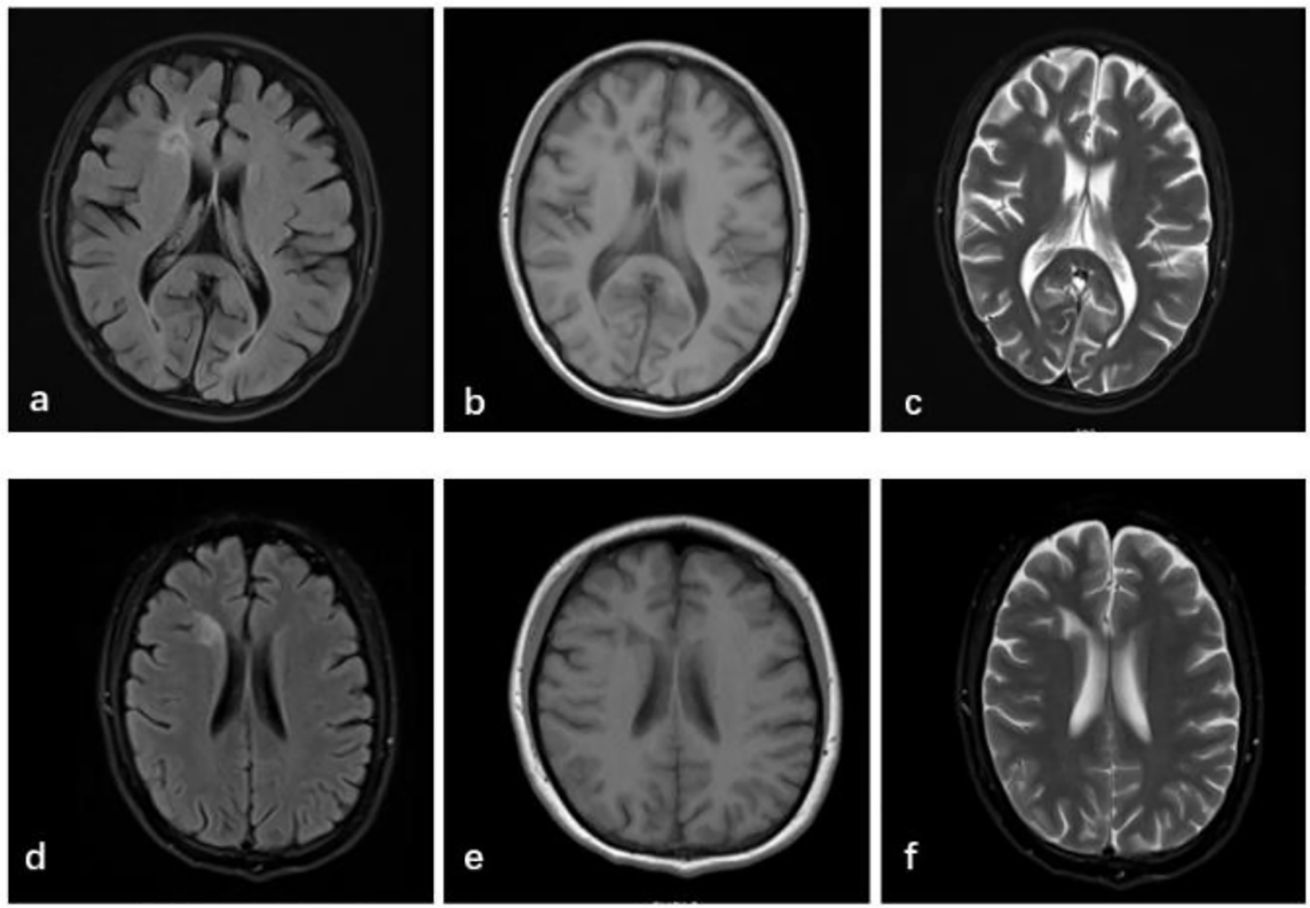
Brain MRI shows white matter lesions in the right anterior horn of the lateral ventricle, with slightly reduced FLAIR signal **(a)** and prolonged T1 **(b)** and T2 **(c)** signal intensities, suggesting focal encephalomalacia with gliosis or demyelination. These findings typically indicate changes after a brain injury, essentially representing that the brain tissue damage that had previous occurred has been resolved and is in a stable state. After 5 months, no new abnormalities were noted compared with prior imaging **(d–f)**.

Repeat laboratory evaluation demonstrated persistent autoantibody positivity (ANA 1:1000, anti-Smith ++, anti-dsDNA ++, anti-nRNP ++, anti-SSA/Ro60 ±, anti-SSB +) and hypocomplementemia (C3: 56mg/dl, C4: 10mg/dl). Hematologic parameters revealed worsening leukopenia (WBC 1.86×10^9^/L), anemia (Hb 96 g/L), and elevated ESR (114 mm/h). The anticardiolipin antibody level was 46.30 RU/mL. Brain MRI revealed a patchy lesion with slightly prolonged T1 and T2 signal intensities in the anterior horn of the right lateral frontal ventricle. FLAIR imaging showed a central hypointense signal with a slightly hyperintense peripheral rim, without restricted diffusion, suggesting a possible area of focal encephalomalacia with gliosis or demyelination (**Figure 1**). A previous study has revealed that the frontal and parietal lobes constitute the most prevalent sites of lesions in NPSLE, with lesions in these areas posing as a risk factor for the development of psychosis (8).

According to the 2019 EULAR/ACR classification criteria for SLE, the patient was diagnosed with SLE. During a period of SLE exacerbation, she experienced acute psychosis, with cranial MRI scans providing evidence for brain injury. Based on the 1997 ACR/Systemic Lupus International Collabo-rating Clinics (SLICC) criteria for neuropsychiatric lupus, she was diagnosed with NPSLE with hematologic involvement and a disease activity score (SLEDAI-2000) of 23. This diagnosis was further supported by psychiatric evaluation.

Although rituximab and cyclophosphamide are commonly used for severe NPSLE, neither was chosen in this case due to financial limitations and concerns about cyclophosphamide toxicity. A literature review identified a case of SLE complicated by rare MOG-antibody disease (MOG-AD), successfully managed with telitacicept, corticosteroids, and immunosuppressants (9). Given the patient’s high autoantibody titers and elevated inflammatory markers, telitacicept was initiated at 160 mg once weekly.

She was concurrently treated with methylprednisolone (500 mg/day for 3 days, tapered to 250 mg/day for 3 days, then 120 mg/day for 3 days, and finally 80 mg/day), mycophenolate mofetil (1 g twice daily), hydroxychloroquine (0.2 g twice daily), and aspirin enteric-coated tablets (0.1 g once daily). Following psychiatric consultation, olanzapine (12.5 mg once nightly), clonazepam (2 mg once nightly), and sustained-release magnesium valproate (0.25 g twice daily) were prescribed. During follow-up, olanzapine was increased to 15 mg, and dexzopiclone (3 mg once nightly) was added.

**Figure 2:**
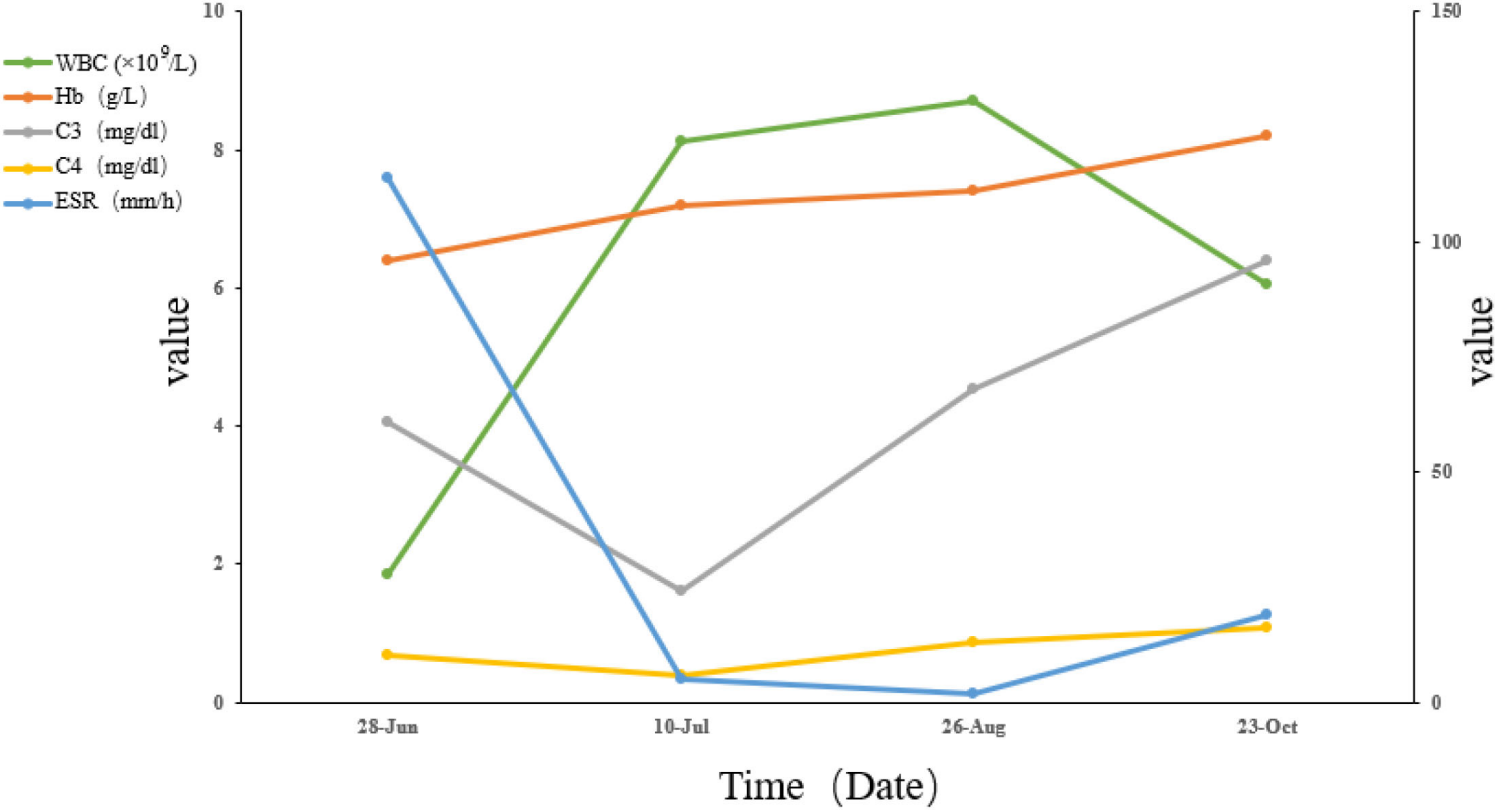
Trends in key laboratory parameters, including white blood cell (WBC), hemoglobin (Hb), erythrocyte sedimentation rate (ESR), complement 3 (C3), and complement 4 (C4). Laboratory reference ranges: WBC: 3.5-9.5 ×10^9^/L; Hb: 115–150 g/L; C3: 79-152mg/dl; C4: 16–38 mg/dl; ESR: 0–26 mm/h.

At the three-month follow-up, laboratory results showed normalization of hemoglobin (123 g/L) and WBC count (6.04×10^9^/L), with improved complement levels (C3: 96mg/dl, C4: 16mg/dl) and ESR reduction to 19 mm/h, as depicted in **Figure 2**. The patient resumed normal daily functioning and independent outpatient visits, with resolution of psychotic symptoms.

**Figure 3:**
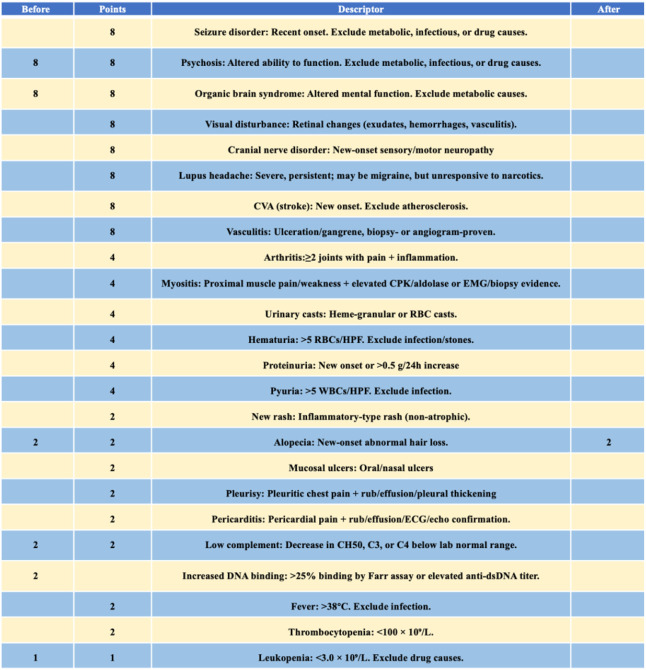
The disease activity score (SLEDAI-2000) decreased from 23 to 2 post-treatment.

**Figure 4:**
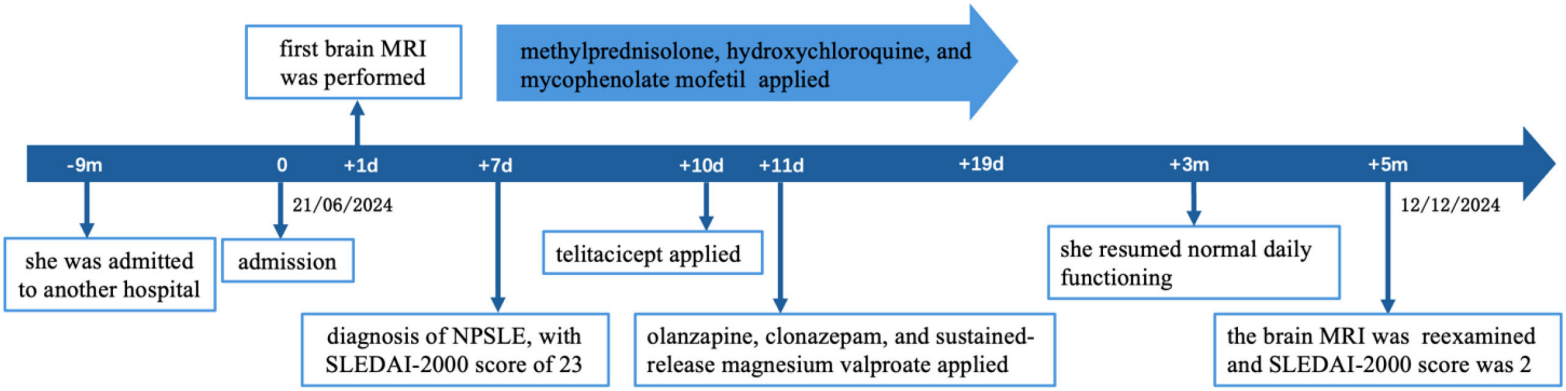
Timeline of the clinical history.

After 5 months, brain MRI revealed persistent white matter lesions in the right anterior horn of the lateral ventricle, with slightly reduced FLAIR signal, suggestive of focal encephalomalacia with gliosis (**Figure 1**). No new abnormalities were noted compared with prior imaging. Her SLEDAI-2000 score had decreased to 2, as shown in **Figure 3**. A visual summary of the treatment timeline is presented in **Figure 4**.

## 3 Discussion

The pathogenesis of NPSLE is primarily mediated through two mechanisms: the autoimmune/inflammatory pathway and the vascular/ischemic/thrombotic pathway (1). A central question in the pathological understanding of NPSLE is whether disruption of the blood-brain barrier (BBB) serves as a pivotal factor (10). While focal NPSLE often results from cerebrovascular involvement, the mechanisms underlying increased BBB permeability in diffuse NPSLE remain unclear (10). Therapeutic approaches generally integrate mechanism-specific interventions, such as immunosuppressants and/or anticoagulants, with symptomatic treatment, such as antipsychotic, antidepressant, and anxiolytic medications. In moderate-to-severe cases, immunosuppressive treatment is typically initiated using glucocorticoids, often in combination with cyclophosphamide or B-cell–directed biologics (11). Mycophenolate mofetil, which selectively inhibits lymphocyte proliferation and suppresses B-cell antibody production, also represents a therapeutic option (12, 13).

Abnormal B-cell activation plays a fundamental role in the pathophysiology of SLE (14). In NPSLE, cytokines from peripheral or central sources can disrupt the BBB, facilitating the entry of autoantibodies and B cells into the central nervous system (CNS). This process contributes to neurotoxicity and glial cell activation through autoantibody deposition (15).

Given the central role of B cells, targeting this pathway has emerged as a promising strategy for NPSLE management. Rituximab, a monoclonal antibody against the CD20 surface antigen on B cells, has shown efficacy in refractory NSPLE, particularly in cases induced by autoantibodies (4).

Another B-cell regulator, the B-cell-activating factor (BAFF or BLyS), belongs to the tumor necrosis factor (TNF) family and is essential for B-cell maturation. Belimumab, a monoclonal antibody targeting BAFF, although not approved specifically for NPSLE, has shown favorable effects on neuropsychiatric symptoms in severe NPSLE (16). In two Phase III trials of belimumab in patients with SLE, improvements in CNS-related symptoms were also observed with belimumab treatment (17). Some researchers suggest that BAFF inhibition may have advantages over CD20-targeted therapies, particularly because BAFF receptors and CD20 differ in their expression on certain cell populations such as plasma cells, although they overlap in many B-cell subsets (18).

APRIL, another key molecule in this pathway, promotes B-cell survival and intrathecal antibody production, thereby aggravate neuronal damage/dysfunction (19). Levels of APRIL in cerebrospinal fluid (CSF) are reportedly 1.5 times higher in patients with NPSLE than in those with SLE without CNS involvement (18). Elevated levels of APRIL in the CSF have also been noted in NPSLE compared with those in other neurological diseases, although further experimental data are needed to clarify APRIL’s role in CNS inflammation (20). Telitacicept, a fusion protein that inhibits both BLyS and APRIL, is approved in China for treating SLE with high disease activity unresponsive to conventional therapy. Its dual-targeting mechanism may offer enhanced safety over CD20-targeted therapies by inducing partial B-cell depletion while potentially avoiding prolonged B-cell depletion (21, 22).

Although glucocorticoids and mycophenolate mofetil have also been suggested to use for the treatment of NPSLE, the overall efficacy of mycophenolate is modest. B-cell-targeted biologics represent a promising therapeutic avenue. In the present case, psychosis related to NPSLE was successfully managed with a combination of telitacicept, corticosteroids, and immunosuppressants (mycophenolate mofetil and hydroxychloroquine). The sustained clinical improvement is likely due to the combined immunomodulatory effects of these agents, in conjunction with antipsychotic medications. The treatment regimen was well tolerated, with no adverse events or infections reported during follow-up. However, further clinical studies are necessary to validate the efficacy and safety of telitacicept in NPSLE management.

## 4 Conclusion

This case suggests that the combination of telitacicept, corticosteroids, and immunosuppressants may offer therapeutic benefit in managing NPSLE, particularly in cases presenting with predominant psychiatric manifestations. Nonetheless, additional studies are needed to substantiate these findings and guide clinical application.

## Data Availability

The original contributions presented in the study are included in the article/**Supplementary Material**. Further inquiries can be directed to the corresponding author.
