# Supplementary material for: Case Report: A patient presenting primarily with psychosis of NPSLE treated with telitacicept, corticosteroids, and immunosuppressants
Source: Front Immunol. 2025 Jul 11;16:1626721. doi: 10.3389/fimmu.2025.1626721 (PMC12289511; doi:10.3389/fimmu.2025.1626721)
Supplement: Supplementary file 1 [file Table1.docx]

Supplementary Material

# Supplementary Table

# During the course of the patient’s disease in the article, the changing process of important laboratory test results (including white blood cell count (WBC), hemoglobin (Hb), erythrocyte sedimentation rate (ESR), Complement 3 (C3) and complement 4 (C4)).

Table 1. The changing process of important laboratory test results.

|  | **June 28** | **July 10** | **August 26** | **October 23** |
| --- | --- | --- | --- | --- |
| **WBC** (×10^9^/L) | 1.86 | 8.13 | 8.7 | 6.04 |
| **Hb**（g/L） | 96 | 108 | 111 | 123 |
| **C3**（g/L） | 0.61 | 0.47 | 0.68 | 0.96 |
| **C4**（g/L） | 0.1 | 0.06 | 0.13 | 0.16 |
| **ESR**（mm/h） | 114 | 5 | 2 | 19 |

Note: Laboratory Reference Range for Indicators: WBC: 3.5-9.5 ×10^9^/L; Hb: 115-150 g/L; C3: 0.79-1.52 g/L; C4: 0.16-0.38 g/L; ESR: 0-26 mm/h.

# Supplementary Figures

# At 5 months of follow-up, the brain MRI was reexamined and there are no significant changes compared to the previous examination.

#
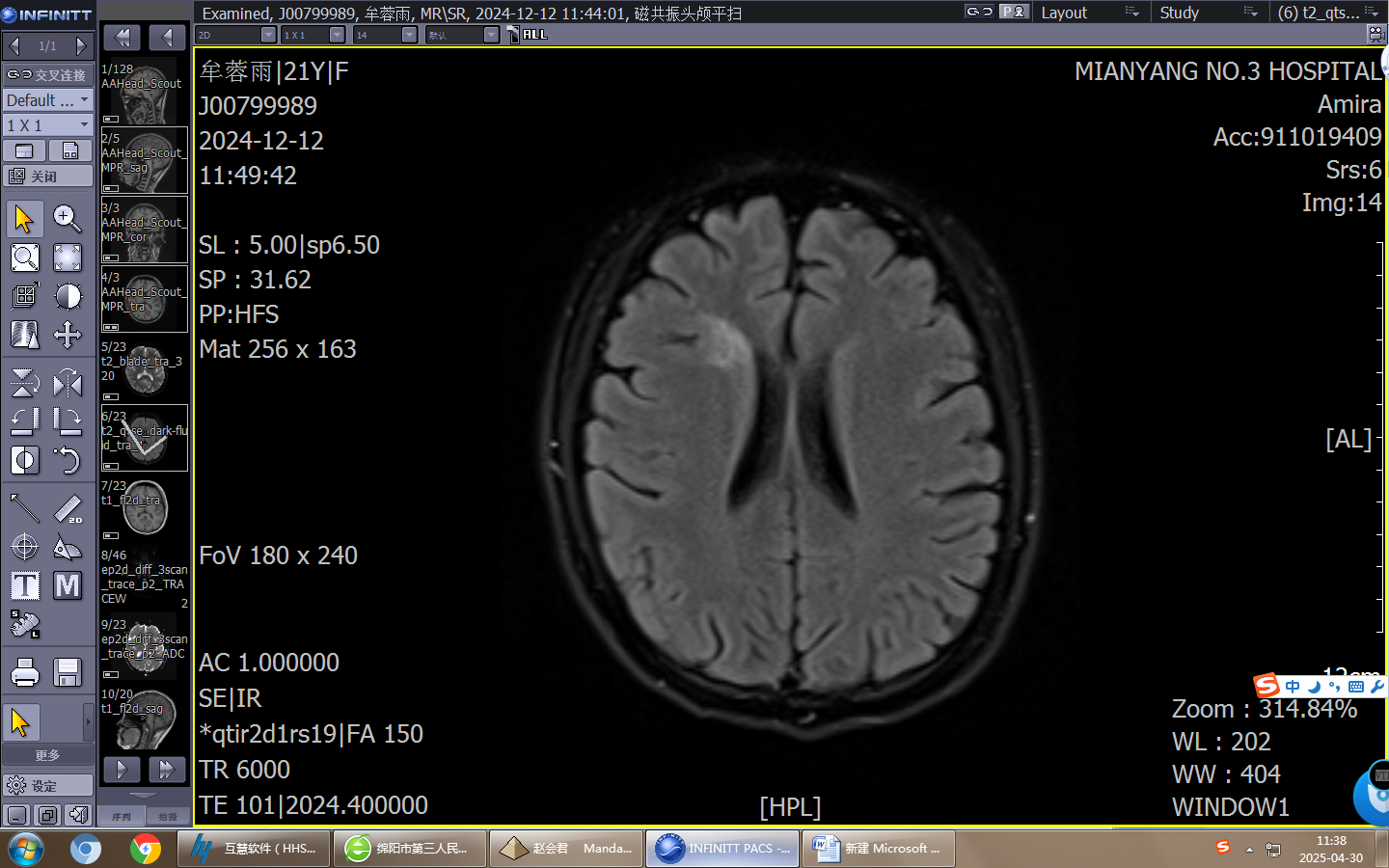


Figure 1. Brain MRI still showing a patchy white matter lesion observed in the right anterior horn of the lateral ventricle.
